# Identification of practitioners at high risk of complaints to health profession regulators

**DOI:** 10.1186/s12913-019-4214-y

**Published:** 2019-06-13

**Authors:** Matthew J. Spittal, Marie M. Bismark, David M. Studdert

**Affiliations:** 1 Melbourne School of Population and Global Health, The University of Melbourne, Parkville, Vic 3010 Australia; 2 Stanford University Medical School and Stanford Law School, Stanford University, 117 Encina Commons, Stanford, CA 94305 USA

**Keywords:** Patient complaints, Quality and safety, Risk prediction, Doctors, Dentists

## Abstract

**Background:**

Some health practitioners pose substantial threats to patient safety, yet early identification of them is notoriously difficult. We aimed to develop an algorithm for use by regulators in prospectively identifying practitioners at high risk of attracting formal complaints about health, conduct or performance issues.

**Methods:**

Using 2011—2016 data from the national regulator of health practitioners in Australia, we conducted a retrospective cohort study of 14 registered health professions. We used recurrent-event survival analysis to estimate the risk of a complaint and used the results of this analysis to develop an algorithm for identifying practitioners at high risk of complaints. We evaluated the algorithm’s discrimination, calibration and predictive properties.

**Results:**

Participants were 715,415 registered health practitioners (55% nurses, 15% doctors, 6% midwives, 5% psychologists, 4% pharmacists, 15% other). The algorithm, PRONE-HP (Predicted Risk of New Event for Health Practitioners), incorporated predictors for sex, age, profession and specialty, number of prior complaints and complaint issue. Discrimination was good (*C*-index = 0·77, 95% CI 0·76–0·77). PRONE-HP’s score values were closely calibrated with risk of a future complaint: practitioners with a score ≤ 4 had a 1% chance of a complaint within 24 months and those with a score ≥ 35 had a higher than 85% chance. Using the 90th percentile of scores within each profession to define “high risk”, the predictive accuracy of PRONE-HP was good for doctors and dentists (PPV = 93·1% and 91·6%, respectively); moderate for chiropractors (PPV = 71·1%), psychologists (PPV = 54·9%), pharmacists (PPV = 39·9%) and podiatrists (PPV = 34·0%); and poor for other professions.

**Conclusions:**

The performance of PRONE-HP in predicting complaint risks varied substantially across professions. It showed particular promise for flagging doctors and dentists at high risk of accruing further complaints. Close review of available information on flagged practitioners may help to identify troubling patterns and imminent risks to patients.

## Background

Despite significant advances internationally in the regulation of health professionals – including requirements to demonstrate ongoing competence, the development of impairment and performance pathways and greater external governance – oversight of fitness of practice remains fundamentally reactive in nature. Practitioner boards rely on reports emanating chiefly from patients, peers, and employers to identify misconduct and impairment. The boards vet those reports, determine which to investigate, and ultimately prosecute a small fraction, with sanctions ranging up to license suspension or revocation [1]. Aspects of this regime are out of step with contemporary approaches to quality assurance and consumer protection [2]. In particular, critics have pointed to the reactive posture of professional boards, their heavy dependence on external actors to observe and report problems, and a paucity of response measures designed to rehabilitate clinicians and prevent future harm [2–5].

In recent years, there has been growing interest among health regulators in approaches to oversight that are more proactive and data-driven [6, 7]. One such strategy involves using administrative data that regulators routinely gather, including demographic and complaint history information to flag practitioners at high risk of future malpractice claims and complaints [8]. The potential for risk prediction to contribute meaningfully to prevention is highlighted by mounting evidence from Australia and the United States that a relatively small number of doctors who recidivate are responsible for a majority of malpractice claims and disciplinary actions [9–12].

Using comprehensive national data on complaints against practitioners in 14 health professions in Australia, we extended an earlier effort aimed at predicting the risk of a complaint at the individual doctor level [8]. Our goal was to test the feasibility of an algorithm that regulators could use to reliably identify high risk practitioners. More generally, we aimed to explore how well simple, routinely-collected administrative data performed in predicting complaint risk and the conditions under which prediction was possible.

## Methods

### Setting

In Australia, all registered health professions are regulated under a national scheme [13]. Ten professions have been regulated by the scheme since its inception in 2010: doctors, nurses and midwives, dental practitioners, psychologists, pharmacists, chiropractors, optometrists, osteopaths, physiotherapists and podiatrists. Four more professions joined in mid-2012: Aboriginal and Torres-Strait Islander (ATSI) health practitioners, Chinese medicine practitioners, medical radiation practitioners and occupational therapists. In 2018, nursing and midwifery were recognised as separate professions and paramedicine joined the scheme.

The Australian Health Practitioner Regulation Agency (AHPRA) works in partnership with practitioner boards that oversee each of the regulated professions. AHPRA manages the registration processes across Australia. In four of the six states and both territories: it receives “notifications” regarding the health, conduct, and performance of registered practitioners; it then refers the notification to the relevant practitioner board for adjudication. (We refer to notifications hereafter as “complaints” for consistency with international terminology.) Two states have slightly different regulatory arrangements. In New South Wales, complaints are managed by the state’s profession-specific boards (e.g. the Medical Council), with support from the Health Professionals Council Authority (HPCA) and the Health Care Complaints Commissioner. In Queensland, since July 2014, the Office of the Health Ombudsman receives complaints about health practitioners and determines whether to manage them internally or transfer them to AHPRA. The divisions of complaint-handling responsibilities among federal and state regulators are described in more detail elsewhere [14].

We conducted the study in accordance with TRIPOD guidelines for developing prediction models [15].

### Data and variables

#### Registration and complaints data

Using administrative data routinely collected by AHPRA, we identified and extracted information on all health practitioners registered to practise in Australia between 1 January 2011 and 31 December 2016. (For the four professions that joined in 2012, the information covered 1 July 2012 to 31 December 2016.) The extracts consisted of variables indicating the period when each practitioner was registered as well as the practitioner’s type of registration, age band, sex, profession, specialty and practice location.

We also identified all complaints about these practitioners lodged with relevant regulators during the same time periods. AHPRA and the HPCA provided practitioner-level complaints data that included the date the complaint was lodged and the primary issue raised by the complainant. We linked the registration data with the complaints data using anonymised, unique identification variables provided by AHPRA and HPCA.

To protect confidentially, APHRA provided practitioners’ birth dates in 5 year bands (e.g., 1970–1974). We recoded these to reflect the practitioner’s age group in 2010. We coded the professions into 16 categories, separating nurses and midwives and distinguishing dentists and dental prosthetists from other allied dental practitioners (dental hygienists, dental therapists and oral health therapists). Within the medical profession we further sub-coded doctors into 10 specialty groups [16] (general practice, surgery, obstetrics and gynaecology, physician, psychiatry, anaesthesia, radiology, emergency and ICU, non-clinical and non-specialist); and sub-coded nurses into registered nurses (who have a three year degree in nursing) or enrolled nurses (who have a two-year diploma or equivalent). If a practitioner had multiple professions (e.g., physiotherapist and nurse) we selected one at random and if they had multiple registration types (e.g., general and specialist) we selected the highest level of registration.

As part of the complaint-handling process, AHPRA and HPCA staff had coded the primary issue associated with each complaint into one of 149 mutually exclusive categories. Using an established coding methodology [1], two reviewers on our team then independently classified these into one of 14 categories. For example, complaints about “bullying, discrimination, disrespect, threats and assault” were re-coded as “interpersonal behaviour”. The independent reviewers’ judgements were then compared and differences resolved by consensus. Finally, the 14 issue categories were arranged into three domains (health, performance and conduct). Further details of the coding process are reported elsewhere [1].

Primary practice location was coded into one of three categories to capture the remoteness of the practice: major city, inner/outer regional area, remote/very remote area. These categories are based on a standard geographic coding system developed by the Australian Bureau of Statistics to indicate proximity to population centres [17].

#### Clinical hours data

Systematic differences among practitioners in the amount of clinical work they conducted had the potential to bias estimates of risk factors for complaints. To control for such differences, we estimated the average number of clinical hours worked per week by profession, sex, age group and for doctors, specialty. Data for these estimates came from the 2015 National Health Workforce Dataset [18], a summary of information practitioners report when renewing their registration each year. Our calculations produced stratified averages of clinical hours per week, which we then merged with the registration data.

#### Study dataset

We constructed a person-period dataset in which each row of data represented covariate values for an individual practitioner for each time interval they were at risk of a complaint. The values for a practitioner’s sex, age group, profession and speciality and practice location did not change over time. The other variables were time-varying. New intervals triggered new rows, which began on the date the value of a time-varying variable changed and ended at the next change of any time-varying variable.

Complaints were also specified as time-varying variables. A practitioner’s “exposure” to complaints was defined in two different ways: (1) a variable indicating the total number of previous complaints (0, 1, 2, 3, 4, 5, 6, ≥7) a practitioner had accumulated; and (2) 14 “look-back” variables (one for each issue category) indicating whether the practitioner had experienced a complaint of that type in the past year. Because construction of the look-back variables necessitated at least one prior year of observation time, we excluded from the analysis each practitioner’s first year of data. For most practitioners, observation time therefore began on 1 January 2012. We also excluded time intervals in which a practitioner was unregistered, was registered to an address outside Australia, held non-practising registration status (which indicates no authorisation to deliver direct clinical care) or was over 75 years of age.

Finally, because our study sought both to develop and validate a risk score for health practitioners, we randomly split the data into a training sample (70% of practitioners) and a validation sample (the remaining 30%).

### Analyses

We began by calculating the number of complaints and the crude complaint rate (per 1000 person years) for the sample as a whole and by practitioner and complaint characteristics. Construction of the complaint risk calculator proceeded in three steps, as described below. All analyses were performed as complete-case analyses (reliance on administrative data meant there was no missing data on any study variables).

#### Predicting complaints

We used survival analysis to identify the characteristics of practitioners at risk of one or more complaints. Specifically, we used an Anderson-Gill model [19], which allows each practitioner to accrue multiple complaints over the study period. The underlying distribution for time at risk was modelled using the Weibull distribution. We calculated cluster-adjusted robust standard errors to account for multiple periods of observation per practitioner. Model discrimination was assessed using the *C*-index derived from fitting the model estimates from the training sample to the validation sample.

Exposure time was defined as the period of time each practitioner was registered with AHPRA (in years) multiplied by weekly clinical hours estimates that corresponded to the practitioner’s profession, sex and age group (and for doctors, specialty). The measure of clinical work time was expressed as a fraction of a 40 h working week and allowed for values greater than 1.

#### Constructing the PRONE-HP score

We used the results from the survival model to design a scoring system. Each risk factor was assigned points; the number of points assigned was scaled directly from the coefficients in the model. Specifically, we multiplied the log hazard ratios for each predictor by 6·0 and then rounded to the closest integer. This transformation permitted all possible point totals to produce a total “risk score” that ranged from 0 to 100. We refer to the scoring system as PRONE-HP (Predicted Risk of New Event for Health Practitioners). The system permits a simple calculation of a practitioner’s risk score each time a new complaint is lodged against him/her.

#### Evaluating the performance of the PRONE-HP score

We assessed the performance of the PRONE-HP score in several ways. First, to determine whether precision was lost in transforming the model coefficients from a survival model to a simple additive scoring system, we computed discrimination using the *C*-index in the validation sample. Second, to assess calibration of the PRONE-HP score (i.e. how closely PRONE-HP scores correspond with out-of-sample complaint risk), we calculated and compared Kaplan-Meier curves for 5 score ranges in the training and validation samples.

Finally, because our aim was to develop a tool for reliably identifying practitioners at high risk of complaints, we defined high risk groups by demarcating practitioners whose risk scores fell above designated “thresholds”. The distribution of scores varied across professions, rendering a single common threshold too crude. Instead we pegged the threshold to a specified percentile (the 90th) *within* each profession. Using this threshold, we calculated the positive predictive value (PPV), negative predictive value (NPV), sensitivity and specificity of the prediction of a new complaint within 2 years for each profession. These calculations used methods appropriate for censored data [20].

#### Sample size calculations

We did not calculate formal sample sizes for this study. Over the period for which data were available, we used data on all complaints against all registered health practitioners in Australia in order to maximise the power and generalisability of study results.

## Results

### Sample characteristics

The sample consisted of 715,415 health practitioners (Table 1). Twenty-two percent were male and 94% were ≤ 65 years. The largest professional group was nurses (55%), followed by doctors (15%), midwives (6%) psychologists (5%) and pharmacists (4%).

**Table 1:** Characteristics of health professionals and complaints

	N	Percent
Characteristics of health professionals	715,415	100
Male sex	160,124	22·4
Age in 2010		
≤ 25 years	45,829	6·4
26–35 years	177,251	24·8
36–45 years	155,270	21·7
46–55 years	154,644	21·6
56–65 years	138,294	19·3
66–75 years	44,127	6·2
Profession and specialty		
Doctor	104,123	14·6
*General practice*	*25,347*	*3·5*
*Surgery*	*6817*	*1·0*
*Obstetrics and gynaecology*	*1956*	*0·3*
*Physician*	*11,310*	*1·6*
*Psychiatry*	*3508*	*0·5*
*Anaesthesia*	*4699*	*0·7*
*Radiology*	*2545*	*0·4*
*Emergency and ICU*	*2479*	*0·3*
*Non-clinical*	*2285*	*0·3*
*Non-specialist*	*43,177*	*6·0*
Nurse	392,447	54·9
*Registered nurse*	*319,912*	*44·7*
*Enrolled nurse*	*72,535*	*10·1*
Midwife	45,014	6·3
Dental practitioners	22,585	3.2
*Dentists/Dental prosthetists*	*18,120*	*2·5*
*Other dental practitioners*	*4465*	*0·6*
Psychologist	34,509	4·8
Pharmacist	30,778	4·3
ATSI practitioners	595	0·1
Chinese medicine practitioners	4766	0·7
Chiropractors	5135	0·7
Medical radiation practitioners	16,103	2·3
Occupational therapist	18,494	2·6
Optometrist	5119	0·7
Osteopath	2102	0·3
Physiotherapist	28,940	4·0
Podiatrists	4705	0·7
Location of practice		
Major city	534,094	74·7
Inner/outer regional	170,797	23·9
Remote/very remote	10,524	1·5
Complaint issue	39,575	100
Health issues	3220	8.1
*Physical heath*	*492*	*1·2*
*Mental health*	*1266*	*3·2*
*Substance use*	*1462*	*3·7*
Conduct issues	13,799	34.9
*Records and reports*	*2293*	*5·8*
*Use or supply of medications*	*1039*	*2·6*
*Honesty*	*520*	*1·3*
*Fees and servicing*	*1094*	*2·8*
*Interpersonal behaviour*	*3669*	*9·3*
*Sexual boundaries*	*1173*	*3·0*
*Compliance with conditions*	*438*	*1·1*
*Other conduct issues*	*3573*	*9·0*
Performance issues	21,420	54.1
*Prescribing or dispensing*	*2335*	*5·9*
*Procedures*	*2290*	*5·8*
*Treatment, communication and other clinical issues*	*16,795*	*42·4*
Unknown	1136	2·9

These practitioners were the subject of 39,575 complaints over the 2,305,763 person-years of follow-up time. Concerns about treatment or other clinical issues were the most common reason for lodging a complaint (42% of complaints), followed by complaints regarding interpersonal behaviour (9%) and other conduct issues (9%).

### Factors associated with risk of complaint

In multivariate survival analysis that adjusted for working hours, all variables in the model were associated with risk of complaint (Table 2). Male practitioners’ complaint risk was 1·5 times that of female practitioners. There was no significant difference in complaint risks for practitioners 26–35 years and those aged ≤25 years, but practitioners in the older age groups had 1·5 to 2·1 times higher risk and it generally increased with age. Compared to practitioners working in major cities, those based in regional Australia had 1·1 times higher complaint risk and those in remote areas had 1·3 times the risk.

**Table 2:** Complaint rates, survival model, and PRONE-HP scoring system

Variable	Number of complaints^1^	Person years^a^	Rate (per 1000 PY)^a^	Model HR (95% CI)^b^	PRONE-HP Score^b^
Sex					
Female	15,119	1,712,922	8·8	Ref·	0
Male	24,456	592,841	41·3	1·5 (1·4–1·5)	2
Age in 2010					
≤ 25 years	404	68,444	5·9	1·0 (0·9–1·2)	0
26–35 years	5021	576,296	8·7	Ref·	0
36–45 years	9050	518,543	17·5	1·5 (1·5–1·6)	3
46–55 years	11,490	559,539	20·5	1·8 (1·7–1·9)	4
56–65 years	9489	474,202	20·0	1·8 (1·7–1·9)	4
66–75 year	4121	108,739	37·9	2·1 (2·0–2·2)	4
Practice location					
Major Cities of Australia	29,960	1,721,291	17·4	Ref·	0
Inner/Outer Regional Australia	9071	550,022	16·5	1·1 (1·1–1·2)	1
Remote/Very Remote Australia	544	34,450	15·8	1·3 (1·2–1·5)	2
Profession/specialty					
Doctor: General practice	8031	106,846	75·2	11·2 (8·7–14·4)	14
Doctor: Surgery	3704	33,082	112·0	13·3 (10·3–17·2)	16
Doctor: Obstetrics and gynaecology	943	7599	124·1	16·2 (12·5–21·1)	17
Doctor: Physician	2377	46,524	51·1	9·3 (7·2–12·1)	13
Doctor: Psychiatry	1656	13,426	123·3	16·1 (12·4–20·8)	17
Doctor: Anaesthesia	507	20,914	24·2	5·1 (3·8–6·7)	10
Doctor: Radiology	365	11,636	31·4	6·2 (4·7–8·2)	11
Doctor: Emergency and ICU	252	9981	25·2	5·3 (4·0–7·1)	10
Doctor: Non-clinical	209	9092	23·0	4·7 (3·4–6·4)	9
Doctor: Non-specialist	3536	142,058	24·9	7·0 (5·5–9·1)	12
Nurse: Registered nurse	6316	1,068,170	5·9	1·8 (1·4–2·3)	4
Nurse: Enrolled nurse	1312	210,213	6·2	1·8 (1·4–2·3)	4
Midwife	439	129,974	3·4	1·0 (0·8–1·3)	0
Dental: Dentists and Dental Prosthetist	3927	60,152	65·3	11·5 (8·9–14·9)	15
Dental: Other dental practitioners	120	12,231	9·8	3·1 (2·2–4·3)	7
Psychologist	2061	90,962	22·7	6·0 (4·7–7·8)	11
Pharmacist	2038	102,477	19·9	5·6 (4·3–7·2)	10
ATSI practitioner	17	983	17·3	4·6 (2·7–7·8)	9
Chinese medicine practitioner	119	9139	13·0	3·0 (2·1–4·2)	7
Chiropractor	447	15,352	29·1	6·5 (4·9–8·6)	11
Medical radiation practitioner	123	39,520	3·1	Ref·	0
Occupational Therapist	149	39,741	3·7	1·5 (1·1–2·0)	2
Optometrist	234	18,053	13·0	3·1 (2·3–4·2)	7
Osteopath	60	6289	9·5	2·6 (1·8–3·9)	6
Physiotherapist	420	86,599	4·8	1·6 (1·2–2·1)	3
Podiatrist	213	14,750	14·4	4·2 (3·1–5·7)	9
Number of prior complaints					
0	27,612	2,226,534	12·4	Ref·	0
1	6837	63,670	107·4	2·6 (2·5–2·8)	6
2	2430	10,681	227·5	4·0 (3·7–4·3)	8
3	1087	2857	380·5	5·1 (4·6–5·6)	10
4	580	994	583·7	6·8 (6·0–7·7)	12
5	328	475	690·1	6·7 (5·7–7·9)	12
6	197	236	836·0	7·9 (6·4–9·6)	12
≥ 7	504	316	1593·4	12·0 (9·8–14·8)	15
Complaint issue (last 12 months)					
Health: Physical health (Ref· = No)	73	327	223·2	1·7 (1·2–2·4)	3
Health: Mental health (Ref· = No)	292	860	339·4	3·3 (2·7–4·0)	7
Health: Substance use (Ref· = No)	360	909	396·0	4·2 (3·5–5·1)	9
Conduct: Records & reports (Ref· = No)	597	1760	339·2	1·6 (1·4–1·7)	3
Conduct: Use or supply of medications (Ref· = No)	247	825	299·5	2·2 (1·9–2·7)	5
Conduct: Honesty (Ref· = No)	156	395	394·4	3·6 (2·6–5·0)	8
Conduct: Fees and servicing (Ref· = No)	328	941	348·5	1·7 (1·4–2·0)	3
Conduct: Interpersonal behaviour (Ref· = No)	886	2865	309·3	1·8 (1·6–2·0)	3
Conduct: Sexual boundaries (Ref· = No)	401	836	479·5	2·4 (2·0–2·8)	5
Conduct: Compliance with conditions (Ref· = No)	164	303	540·8	1·4 (1·1–1·9)	2
Conduct: Other conduct issues (Ref· = No)	786	2675	293·8	2·0 (1·8–2·3)	4
Performance: Prescribing or dispensing (Ref· = No)	417	1750	238·3	1·6 (1·4–1·9)	3
Performance: Procedures (Ref· = No)	665	1859	357·6	1·5 (1·4–1·7)	3
Performance: Treatment, communication and other clinical issues (Ref· = No)	3110	13,444	231·3	1·4 (1·3–1·5)	2
Survival model, intercept (95% C)				0·0020 (0·0015–0·0025)	
Survival model, shape parameter *p* (95% CI)				0·9616 (0·9498–0·9736)	
*C*-index (95% CI)				0·77 (0·76–0·78)	0·77 (0·76–0·77)

^a^Calculated using the whole sample

^b^Calculated using the training sample (randomly selected 70% of practitioners)

Risks varied widely by profession and medical specialty. Compared to medical radiation practitioners (the profession with the lowest risk and the reference group), the risk of a complaint was substantially higher for all the medical specialties, especially obstetrics and gynaecology (HR = 16·2), psychiatry (HR = 16·1), surgery (HR = 13·3) and general practice (HR = 11·2). Dentists and dental prosthetists were also at very high risk (HR = 11·5). Chiropractors (HR = 6·5), psychologists (HR = 6·0), and pharmacists (HR = 5·6) had elevated risks of complaints, whereas the risks for enrolled nurses (HR = 1·8), registered nurses (HR = 1·8), physiotherapists (HR = 1·6), occupational therapists (HR = 1·5) and midwives (HR = 1·0), were relatively close to those of the reference group.

Complaint risk increased monotonically with the number of prior complaints. Compared to those with no prior complaints, practitioners with 1 prior complaint had 2·6 times higher risk of accruing another complaint; those with 3 prior complaints had 5·1 times the risk and those with ≥7 prior complaints had 12·0 times the risk. The highest risks of additional complaints followed complaints relating to concerns about substance use (HR = 4·2), honesty (HR = 3·6), a practitioner’s mental health (HR = 3·3), sexual boundaries (HR = 2·4) and use and supply of medications (HR = 2·2).

### Performance of PRONE-HP

The far right column of Table 2 shows the points assigned to each predictor, based on the hazard ratios estimated in the survival model, to form the PRONE-HP scoring system. PRONE-HP demonstrated good discrimination when applied to the validation dataset (*C*-index = 0·77). The *C*-index was virtually identical to that estimated in the underlying model, suggesting that the conversion of model coefficients to the points system did not materially affect discrimination.

**Fig. 1:**
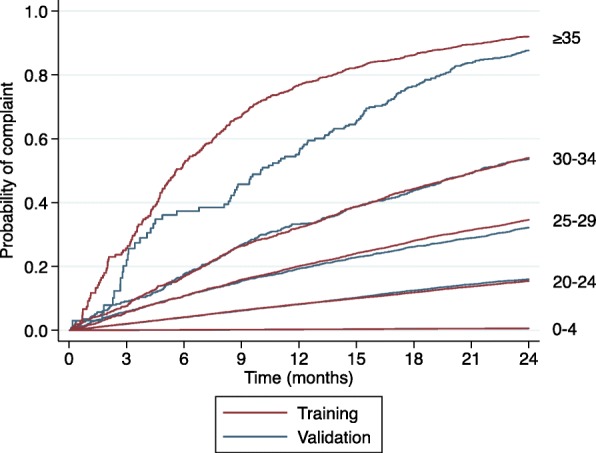
Observed probability of complaints based on selected PRONE-HP score ranges, test and validation samples

To assess the calibration of PRONE-HP, we examined the out-of-sample consistency within risk strata. Figure 1 shows Kaplan-Meier curves plotting the probability of a subsequent complaint over a two-year period 5 different groups of practitioners. The groups are defined by ranges (or bands) of PRONE-HP scores. Within each band, two curves are displayed: one comes from the training dataset and the other from the validation dataset. The pairs of curves in the lowest four ranges show close concordance. In the highest range (PRONE-HP ≥35), there is some divergence in the pair, especially between 6 and 18 months.

Figure 1 also shows a high degree of consistency up and down the PRONE-HP scale. The probability of a subsequent complaint ascends monotonically across the ranges. For example, at 24 months, the probability of a further complaint is 0·6% for practitioners in the 0–4 point range, 15% for practitioners in the 20–24 point range, 30–33% for practitioners in the 25–29 point range, 53–54% for scores 30–34, and 88–92% for scores ≥35. In addition, after the first few months, no group’s pair of prediction curves crossed those of the adjacent group.

### Thresholds for identifying high risk practitioners

For doctors and dentists and dental prosthetists, the high-risk threshold (90th percentile of risk) was a score of 30 or more points (Table 3). A total of 6245 doctors reached or exceeded this threshold at some period during the study. During those periods, the PRONE-HP had a PPV of 93·1% and a NPV of 90·1% for these practitioners. Sensitivity was low (17·6%) but specificity was high (99·8%). The PRONE -HP demonstrated similar levels of predictive accuracy among dentists and dental prosthetists at out above their high-risk threshold. The high PPVs estimated for doctors and dentists and dental prosthetists, coupled with the relatively small number of practitioners exceeding the threshold, suggests that PRONE-HP may be a useful tool for identifying high risk practitioners in these professions.

**Table 3:** Diagnostic properties of PRONE-HP: Predicting new complaint within 2 years

	Threshold (90th percentile)^a^	PPV^b^	NPV^c^	Sensitivity^d^	Specificity^e^	Number of practitioners above threshold^f^
Doctors	30	93·1%	90·1%	17·6%	99·8%	6245
Dentists/Dental Prosthetists	30	91·6%	87·9%	15·3%	99·8%	1203
Chiropractors	23	71·1%	95·7%	23·1%	99·5%	349
Psychologists	18	54·9%	96·8%	32·9%	98·7%	2594
Pharmacists	18	39·9%	96·6%	24·3%	98·3%	1981
Podiatrists	16	34·0%	97·9%	29·6%	98·3%	311
ATSI health practitioners	17	25·3%	97·0%	8·6%	99·2%	50
Optometrists	14	12·7%	97·3%	10·0%	97·9%	553
Osteopaths	12	8·4%	98·7%	29·6%	94·4%	450
Other dental practitioners	12	7·3%	98·5%	9·9%	98·0%	621
Physiotherapists	9	7·3%	99·2%	17·6%	97·7%	2676
Nurses	9	5·7%	99·1%	33·9%	92·8%	95,722
Medical radiation practitioners	6	5·5%	99·6%	30·3%	97·0%	1970
Occupational therapists	7	5·4%	99·3%	19·4%	97·3%	1803
Midwives	5	5·2%	99·5%	27·8%	96·4%	11,581
Chinese medicine practitioners	13	4·7%	98·0%	12·5%	94·3%	1456

^a^Calculated using the person-period dataset in which an individual’s PRONE-HP score can change over time

^b^Positive predictive value: the proportion of those who test positive who have another complaint within 2 years

^c^Negative predictive value: the proportion of those who test negative who do not have another complaint within 2 years

^d^The proportion who test positive among those who have another complaint within 2 years

^e^The proportion who test negative among those who do not have another complaint within 2 years

^f^Calculated using the individual as the unit of analysis (person dataset), taking the maximum PRONE-HP score that was observed

By contrast, 10 of the 16 professions we analysed exhibited features that constrained the practical utility of PRONE-HP. The high-risk threshold of these professions fell at a substantially lower scores and PPVs were low. The professions were Aboriginal and Torres Strait Islander health practitioners (PPV = 25·3%), optometrists (PPV = 12·7%), osteopaths (PPV = 8·4%), other dental practitioners (PPV = 7·3%), physiotherapists (PPV = 7·3%), nurses (PPV = 5·7%), medical radiation practitioners (PPV = 5·5%), occupational therapists (PPV = 5·4%), midwives (PPV = 5·2%) and Chinese medicine practitioners (4·7%). In addition, the number of practitioners in the high-risk groups for nurses and midwives was very large.

In between are several professional groups for whom PRONE-HP may be useful for identifying high risk practitioners so long as regulators are willing to accept lower predictive properties (i.e. a greater number of false positives). Chiropractors (PPV = 71·1%), psychologists (PPV = 54·9%), pharmacists (PPV = 39·9%) and podiatrists (PPV = 34·0%) constitute this group.

## Discussion

In this national study of complaints to health regulators over a 6-year period, we modelled the risk of complaints to the professional regulator among more than 700,000 practitioners in 14 health professions. Results from the model formed the basis of PRONE-HP, a tool designed to be amenable for use by regulators to estimate the risk practitioners’ will experience complaints in the short to medium term. Tests of PRONE-HP’s predictive accuracy suggested it is a very promising approach for identifying doctors and dentists at high risk of subsequent complaints (few false positives); moderately promising for identifying chiropractors, psychologists, pharmacists and podiatrists at high risk (some false positives); and of limited utility for the other professions examined (many false positives).

We envision the PRONE-HP score being used in a couple of different ways by health profession regulators in the complaint handling process. Because the score can be re-calculated each time a complaint is lodged, it could be useful for flagging cases that warrant deeper review – for example, reviewing previous complaints against a practitioner or other information held by the regulator to ascertain if there are troubling patterns of care or imminent risks to patients. Such signposting may be the most appropriate way to use for the tool. Alternatively, or in addition, the score could guide stepped-level interventions. For example, a low PRONE-HP score may suggest that minimal action is required beyond resolution of the immediate complaint, while a high score may prompt a regulator to consider whether a more active intervention is needed to guard against the risk of future harm.

Using the PRONE-HP in this way has the potential to benefit both patients and practitioners. The tool paves the way for targeted quality-improvement interventions. Patients clearly stand to benefit from such risk remediation. Perhaps less obvious is the fact that practitioners may too. By enabling interventions to occur at an early stage among practitioners who need them, before poor track records of medico-legal actions materialise, the tool could help facilitate an environment in which interventions can be constructive and non-punitive in nature. In other words, the anticipatory nature of the predictive tool creates opportunities to mount interventions designed to help practitioners deliver better care, rather than penalising them for damage done [21]. Ensuring that implementation of an instrument like PRONE-HP strikes the right balance between protecting patients and treating practitioners fairly will be challenging but is crucial.

A more general contribution of our study is to clarify the conditions under which prediction of medico-legal events is possible. PRONE-HP’s performance varied widely across professions. In theory, it should perform well under conditions in which complaints cluster among certain individuals and those individuals have observable traits that differentiate them from their peers. In practice, however, there is a more fundamental requirement: clustering, even among clinicians who are relatively prone to behaviours that prompt complaints, depends on a sufficiently high baseline incidence of complaints [22–24]. It is no coincidence that PRONE-HP performed best among professions with the highest complaint rates, and worst among professions with the lowest complaint rates. In sum, the rarity of complaints in certain professions appears to impede robust prediction in those professions.

Our approach differs from previous attempts to predict medico-legal risk (including our own) in several ways. First, we analysed complaint risk in 16 different health profession groups Australia-wide; previous work has focused on samples of doctors, often sampled from particular insurers or states [12, 25–30]. Doctors play a central role in the delivery of healthcare, but many other types of clinicians influence the quality and safety of care patients receive.

Second, we used a wider range of predictor variables than previous studies have – most notably, information about issues raised in prior complaints. This enabled us to identify and account for particular types of complaints – for example, those relating to mental health, substance use, sexual boundaries and honesty – that appear to have very strong associations with recurrence. We have previously shown that some of these types of complaints, most notably mental health and substance use problem, are more likely to end in disciplinary action being taken [1]. Our finding here is that these same issues are also risk factors for recurrent complaints further underlines their indicative value.

Third, the model used to create PRONE-HP accounted for systematic differences in degrees of clinical contact (or “exposure” to complaints) among different practitioner groups. Most previous studies rely on head counts of registered practitioners, which tend to overstate attribution of risk of certain characteristics (e.g. male) that are also associated with higher clinical workloads.

Fourth, while many previous studies of medico-legal risk “fix” characteristics at baseline levels, we allowed several predictors to be time-varying. This allows PRONE-HP to take into account aspects of a practitioner’s risk profile that changed over time.

Finally, our previous studies predicting medico-legal risk have been restricted to practitioners with a least one prior complaint or malpractice claim. [8–10] The risk measures in this study take into account the entire population of registered practitioners, including those with no previous complaints. This full cohort approach enhances statistical power and generalisability, and supports estimation of risk that is related to an entire health workforce.

Notwithstanding these substantial methodological advances over prior work, our analyses has several limitations. First, we were not able to measure certain practitioner-level variables that are known to be related to complaint risk. These include patient volume, practice type, disciplinary history, and for doctors – performance issues during training and country of training.

Second, the issue-type variables in our analysis were coded from information available at the time the complaint was lodged, and subsequent investigation may have uncovered new or different issues. More refined measurement of this variable may have slightly boosted the model’s predictive power.

Third, while complaints are increasingly recognised as a potential marker of potential problems in care, not all complaints are associated with poor performance or wrongdoing [25]. Fourth, the reliance on administrative data means some practitioner’s practice arrangements are incompletely observed. For instance, we rely on the practitioner’s principle practice location to measure remoteness, but some practitioners do not have a fixed practice location or may work across state/jurisdictional boundaries.

Fifth, our data treats each complaint as a separate and independent, but occasionally a single complaint generates multiple complaints and our data did not allow us to link them. Sixth, we do not have a measure of the severity of harms associated with the complaints in our sample. Those harms will have ranged from minor misunderstandings to preventable deaths, but there is currently no established taxonomy among regulators for measuring and grading harm.

Sixth, while we able to obtain high PPV and NPV values for doctors and dentists, the sensitivity of PRONE-HP for all professions, but especially these two, was low. This result stemmed from a decision to set thresholds that maximised PPV, which is particularly relevant and important for a prediction tool of this kind. Finally, at the request of AHPRA, which has oversight responsibility for all regulated professions, we developed a single algorithm for predicting risk across the workforce as whole. Profession-specific algorithms may well perform better, at least among professions within which risk prediction is feasible.

## Conclusions

Some complaints about health practitioners may reflect an isolated instance of dissatisfaction with care; others herald persistent problems that adversely affect the practitioner’s fitness to treat patients [21, 31]. Telling one from the other, without launching time- and resource-intensive investigation, has long been challenge for regulators. The capacity of tools like PRONE-HP to harness routinely-collected information to reliably predict risk of future complaints in some professions opens up new opportunities for regulators of the health professions. Prediction alone is not quality improvement. But it has considerable potential to inform larger quality improvement initiatives and to enable regulators to be more proactive in protecting patients in ways that optimise scarce regulatory resources [6, 7]. Hence, when a prediction capability is evident, as it is for complaint-prone doctors and dentists in Australia, the focus should turn to the nature and effectiveness of the interventions that risk prediction will facilitate.

## Data Availability

The data that support the research findings are owned by AHPRA and HPCA. Restrictions apply to the availability of these data, which were used under license for the current study and so are not publicly available. Other researchers can apply to AHPRA and HPCA for access to the data. The authors are willing to make Stata code used in the study to construct variables and undertake analyses available to other interested researchers.

## Abbreviations

AHPRA: Australian Health Practitioner Regulation Agency
CI: Confidence Interval
HPCA: Health Professionals Council Authority
HR: Hazard Ratio
NPV: Negative Predicative Value
PPV: Positive Predictive Value
PRONE-HP: Predicted Risk of New Event for Health Practitioners
